# A coexistence theory in microbial communities

**DOI:** 10.1098/rsos.180476

**Published:** 2018-09-19

**Authors:** Marina Dohi, Akihiko Mougi

**Affiliations:** Department of Biological Science, Faculty of Life and Environmental Science, Shimane University, 1060 Nishikawatsu-cho, Matsue 690–8504, Japan

**Keywords:** microbial community, bistability, pH, indirect interaction, resilience, mathematical model

## Abstract

Microbes are widespread in natural ecosystems where they create complex communities. Understanding the functions and dynamics of such microbial communities is a very important theme not only for ecology but also for humankind because microbes can play major roles in our health. Yet, it remains unclear how such complex ecosystems are maintained. Here, we present a simple theory on the dynamics of a microbial community. Bacteria preferring a particular pH in their environment indirectly inhibit the growth of the other types of bacteria by changing the pH to their optimum value. This pH-driven interaction always causes a state of bistability involving different types of bacteria that can be more or less abundant. Furthermore, a moderate abundance ratio of different types of bacteria can confer enhanced resilience to a specific equilibrium state, particularly when a trade-off relationship exists between growth and the ability of bacteria to change the pH of their environment. These results suggest that the balance of the composition of microbiota plays a critical role in maintaining microbial communities.

## 1. Introduction

Microbes living in ecosystems create microbial communities and can play key roles in ecosystem functioning [1–5]. They are also potentially critical for our health [6] because of their functions associated with metabolism and immunity [7–10].

Although the composition and functional properties of microbiota have been identified by numerous empirical studies [1–5,11], we possess an insufficient understanding of how complex microbial communities are maintained or destroyed [12–19]. For example, a key question is whether compositional changes in the microbiota are caused by abrupt changes in alternative stable states [15,20], as suggested by empirical studies [21–27]. Another key question is how the composition of microbiota is maintained. Here, we present a simple mathematical model to answer these questions.

We consider a fundamental interaction among bacteria involving a pH-driven indirect interaction. The pH of the bacterial environment, which is changed by the bacteria themselves [28], critically affects their growth [29–32]. Consider two different types of bacteria in terms of pH change and preference. An acidophilic bacterium such as *Bifidobacterium* and an alkaliphilic bacterium such as *Clostridium perfringens* prefer acid or alkaline environments, respectively [30,31], and sensitivity to pH critically affects their growth. Bacteria change the pH of the environment to optimum values through the production of acid or alkaline compounds [28]. Here, we used a dynamic, analytical model of acidophilic and alkaliphilic bacteria and pH to show that pH-driven indirect interactions between two functional groups of bacteria always caused a state of bistability in which either group dominated. Furthermore, over a range of parameters, a moderate abundance ratio of different bacteria led to the highest resilience of more abundant bacteria at the equilibrium. There was a tendency towards high resilience when there was a trade-off between growth and the rates of change of pH. The composition of the microbiota may therefore play a critical role in maintaining the ecosystems.

## 2. Model

Consider an ecosystem comprising two types (functional groups) of bacteria that indirectly interact through changes in pH caused by each. Low and high pH are suitable environments for acidophilic and alkaliphilic bacteria, which can change pH, and decrease and increase pH, respectively. The simplest ecosystem model based on this scenario is defined by the following ordinary differential equations:

2.1*a* $$\frac{\text{d}X_{1}}{\text{d}t}=\left\{r_{1}\left(\frac{1}{1+e^{\theta Y}}\right)-X_{1}\right\}X_{1},$$

2.1*b* $$\frac{\text{d}X_{2}}{\text{d}t}=\left\{r_{2}\left(\frac{1}{1+e^{-\theta Y}}\right)-X_{2}\right\}X_{2}$$

2.1*c* $$\text{and}\;\frac{\text{d}Y}{\text{d}t}=(a_{2}X_{2}-a_{1}X_{1})(1-Y^{2}),$$

where *X*_1_, *X*_2_ and *Y* represent the population sizes of acidophilic bacteria and alkaliphilic bacteria and pH, respectively. The variable *r_i_* represents the maximum growth rate of each bacterial population, which is normalized as a function of the strength of self-regulation without loss of generality; *θ* represents the sensitivity parameter of the effects of pH on bacterial growth (pH sensitivity); and *a_i_* represents the rate of pH change caused by the bacteria. Note that pH is also self-regulated for avoiding divergence. However, many other species and their interactions [26] within each functional group are not considered here. Although the substrates of each bacterium differ as assumed here, the acidophilic and alkaliphilic bacteria are the major consumers of carbohydrates and protein, respectively [28], within each group, complex interactions (e.g. competition versus cooperation) may exist among species [33]. Our model does not explicitly consider such complicated species interactions. However, through self-regulation and changes in pH, the model may capture the essential properties of competition and cooperation operating within a functional group.

## 3. Results

**Figure 1.**
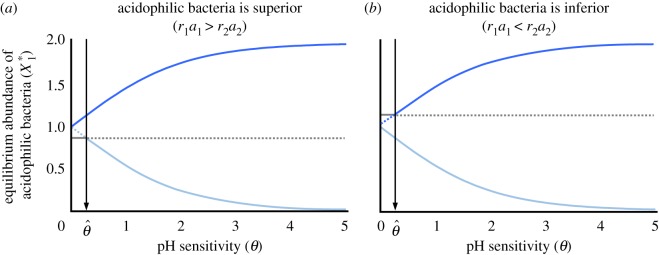
Equilibrium abundance of acidophilic bacteria varies with sensitivity to pH. (*a*) *r*_1_*a*_1_ > *r*_2_*a*_2_. (*b*) *r*_1_*a*_1_ < *r*_2_*a*_2_. The different colours indicate three equilibria. Dotted and solid lines indicate locally unstable and stable equilibria, respectively. The arrows indicate the threshold value of *θ* at which the stability shifts $\left(\hat{\theta }\right)$. In (*a*), *r*_1_ = 2, *a*_1_ = 1.3, *r*_2_ = 2 and *a*_2_ = 1. In (*b*), *r*_1_ = 2, *a*_1_ = 1, *r*_2_ = 2 and *a*_2_ = 1.3.

The nature of pH sensitivity invariably causes bistability (figure 1). The system has three equilibria (*X*_1_*, *X*_2_*, Y*): (i) (*r*_1_/(1 + *e*^−*θ*^), *r*_2_/(1 + *e^θ^*), −1), (ii) (*r*_1_/(1 + *e^θ^*), *r*_2_/(1 + *e^−θ^*), 1) and (iii) (*a*_2_*r*_1_*r*_2_/(*a*_1_*r*_1_ + *a*_2_*r*_2_), *a*_1_*r*_1_*r*_2_/(*a*_1_*r*_1_ + *a*_2_*r*_2_), ln[*r*_1_*a*_1_/*r*_2_*a*_2_]/*θ*). The stability of three equilibria can change as a function of the strength of pH sensitivity *θ* (see electronic supplementary material, S1). When acidophilic bacteria are superior (*r*_1_*a*_1_ > *r*_2_*a*_2_), the first equilibrium is always locally stable regardless of *θ* (figure 1*a*). The second equilibrium is locally stable when pH sensitivity is greater than the threshold $\hat{\theta }$ (=ln[*r*_1_*a*_1_/*r*_2_*a*_2_] > 0). By contrast, when acidophilic bacteria are inferior (*r*_1_*a*_1_ < *r*_2_*a*_2_), the second equilibrium is always locally stable regardless of *θ*, whereas the first equilibrium is locally stable when $\theta >-\hat{\theta }$(figure 1*b*). Unlike these equilibria, the stability of the third equilibrium is independent of the superiority of acidophilic bacteria (figure 1); rather, it is locally stable when $\theta <\hat{\theta }$. Thus, when pH sensitivity is high $\left(\theta >\hat{\theta }\right)$, the more abundant (first) and less abundant (second) equilibria of acidophilic bacteria are locally stable. By contrast, when pH sensitivity is low $\left(\theta <\hat{\theta }\right)$, either one of first and second equilibria becomes unstable depending on the superiority of bacteria, and instead, the third equilibrium becomes stable.

Using numerical simulations, we found that the dynamics converged to either equilibrium, depending on initial abundance (electronic supplementary material, figure S1 in S2). However, the convergence to the third equilibrium was difficult because the domain of attraction for the third equilibrium in the case of $\theta <\hat{\theta }$ was limited. Therefore, the trajectory for *X_i_* usually approaches either of the other two equilibria. These results suggest that the system tended to reach equilibrium (first more abundant equilibrium of acidophilic bacteria when *r*_1_*a*_1_ > *r*_2_*a*_2_ and second less abundant when *r*_1_*a*_1_ < *r*_2_*a*_2_) when pH sensitivity was low $\left(\theta <\hat{\theta }\right)$, while it tended to converge on one equilibrium, depending on the initial condition when pH sensitivity was large $\left(\theta >\hat{\theta }\right)$.

**Figure 2.**
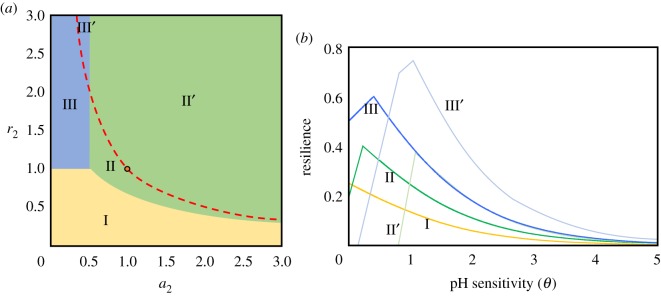
Relationships between pH sensitivity and resilience. (*a*) Phase diagram of three cases classified according to the shifts of dominant eigenvalues with pH sensitivity (electronic supplementary material, S1). We assumed *r*_1_ = *a*_1_ = 1 (orange circle). The red line in (*a*) separates the two patterns of stability shift, as shown in figure 1, in the upper area, *r*_1_*a*_1_ < *r*_2_*a*_2_, and lower area, *r*_1_*a*_1_ > *r*_2_*a*_2_. Hence, II’ and III’ include the unstable regions in (*b*) (lower values of *θ*). In (*b*), typical cases of relationships between pH sensitivity and resilience. In I, resilience monotonically decreases as a function of *θ.* In II (II′) and III (III′), resilience peaks at an intermediate value of *θ*. II (II′) and III (III′) have different ratios of *X*_1_*/*X*_2_* at the peaks of resilience, each of which are 1 and (2*a*_2_ + 1)/2*a*_1_, respectively (electronic supplementary material, S1). Parameter values (*r*_2_, *a*_2_) of I, II, II′, III and III′ in (*b*) are as follows: (0.5, 0.5), (0.9, 0.9), (1.5, 1.5), (1.5, 0.2) and (2.9, 0.4), respectively.

**Figure 3.**
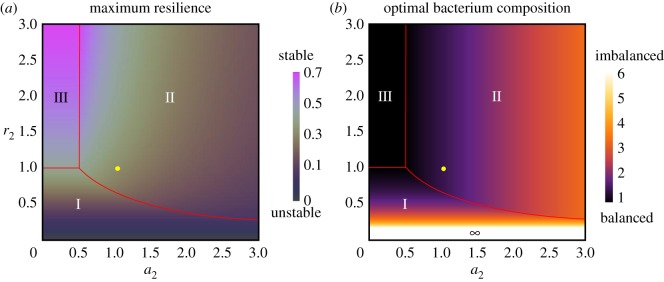
Maximum resilience *R*_max_ (*a*) and optimum microbial composition *X*_opt_ (*b*) in a focusing equilibrium. We assumed *r*_1_ = *a*_1_ = 1. Contours in (a) indicate the values of *R*_max_ in phases I, II and III, which are equal to *r*_2_/2, 2*a*_1_*r*_1_*r*_2_/(2*a*_2_*r*_2_ + *r*_2_ + 2*a*_1_*r*_1_) and *r*_2_*r*_1_/(*r*_2_ + *r*_1_), respectively (electronic supplementary material, S1). Contours in (*b*) indicate the values of *X*_opt_ in phases I, II and III, which are equal to *r*_1_/*r*_2_ (2*a*_2_ + 1)/2*a*_1_ and 1, respectively (electronic supplementary material, S1). In the region with lower values of *r*_2_ in (*b*), *X*_opt_ has a much higher value. Parameter values are the same as those shown in figure 2*a*. The position of *r*_1_ = *a*_1_ (=1) is indicated by the yellow circles.

Once the system achieves a stable equilibrium, how is it maintained or changed to an alternative state? To answer this question, we evaluated the stability of the equilibrium according to resilience, the capacity of the system to return to a stable equilibrium after encountering a short and small disturbance, measured by the absolute value of the highest real part of eigenvalues of the Jacobian matrix. Here, consider a situation in which acidophilic bacteria are in the more abundant (first) equilibrium (or alkaliphilic bacteria are in the less abundant equilibrium). When we calculated the resilience of the equilibrium dominated by acidophilic bacteria (electronic supplementary material, S1), pH sensitivity greatly affected resilience. Two main mechanisms explain how pH sensitivity changes resilience. The resilience monotonically decreased as a function of the increase in pH sensitivity or peaked at an intermediate level of pH sensitivity (figure 2*b*). The key factors that determined these patterns were the superiority of growth and the rates of change of pH. When acidophilic bacteria were superior (*r*_1_ > *r*_2_, *a*_1_ > *a*_2_), the former was likely to occur, otherwise the latter was likely (electronic supplementary material, S1 and figure 2*a*). We found that the peak resilience (*R*_max_) tended to be high when growth and rates of changing pH tended to exist in a trade-off relationship (*r*_1_ < *r*_2_, *a*_1_ > *a*_2_; see electronic supplementary material, S1 and figure 3*a*).

Furthermore, we found that resilience was related to the compositions of the microbiota. The ratio of the equilibrium abundance (*X*_1_*/*X*_2_*, where X_i_* is the equilibrium abundance of each bacterial population) of acidophilic bacteria to alkaliphilic bacteria exponentially increased with an increase in pH sensitivity (*e^θ^r*_1_/*r*_2_; see electronic supplementary material, S1). This generally suggests that the imbalance of microbiota compositions (large difference in equilibrium abundances of the bacteria) can decrease resilience. However, when the resilience peaked at an intermediate level of pH sensitivity, a balance of the compositions of microbiota (small difference in equilibrium abundances of the bacteria) greatly increased resilience. Particularly, when growth and the rates of pH change tended to exist in a trade-off relationship (*r*_1_ < *r*_2_, *a*_1_ > *a*_2_; electronic supplementary material, S1 and III in figure 3*a* and *b*), the resilience reached a maximum at a perfect balance of composition (*X*_1_*/*X*_2_* = 1). By contrast, when alkaliphilic bacteria tended to be superior (*r*_1_ < *r*_2_, *a*_1_ < *a*_2_; II in figure 3*b*), the optimal composition maximizing resilience (*X*_opt_ defined as *X*_1_*/*X*_2_* maximizing the resilience) was a function of the rate of change of pH (2*a*_2_ + 1)/2*a*_1_ (electronic supplementary material, S1). This outcome required condition *a*_1_ − *a*_2_ < 1/2 (electronic supplementary material, S1), indicating that the optimal composition *X*_opt_ was > 1. Moreover, a relatively balanced composition (*X*_opt_ < 3) maximized the resilience over a broad parameter space (electronic supplementary material, figure S2 in S2). These results suggest that a balanced microbial composition plays a major role in self-maintenance, particularly when growth and the rate of pH change exist as a trade-off relationship (figure 3).

A sigmoidal functional form was used in the pH effect for bacterial growth for analytical simplicity. This implies that there were no negative consequences on bacteria populations at both extremes of pH. However, alkaline or acidic microenvironments do not always have a positive effect on bacterial growth rates. Acidophilic (or alkaliphilic) bacteria can drive the pH so low (or high) that it begins to negatively affect its own population [34,35]. Here, we consider another functional form describing such negative effects to examine the robustness of the results. We used a bell-shaped function for bacterial growth [34], $r_{i}{\text{e}}^{-{\left(Y-p_{i}\right)}^{2}/\sigma^{2}}$, where *p_i_* is the optimal pH for each bacteria (*p*_1_ < 0 and *p*_2_ > 0) and *σ* is the pH sensitivity parameter. Even in this model, we can demonstrate how the main results are qualitatively held. First, bistability occurs, which is followed by the coexistence of two non-trivial equilibria, the acidic (Y* = −1) and alkaline (Y* = 1) equilibria (see electronic supplementary material, S1, for details of *X*_1_* and *X*_2_*). When ln[*r*_2_*a*_2_/*r*_1_*a*_1_] < (1 + *p*_1_)^2^/*σ*^4^ + (1 + *p*_2_)^2^/*σ*^2^, the acidic equilibrium is locally stable. When acidophilic bacteria are superior (*r*_1_*a*_1_ > *r*_2_*a*_2_), the acidic equilibrium is stable regardless of *σ* (electronic supplementary material, figure S4 in S2). Conversely, when acidophilic bacteria are inferior (*r*_1_*a*_1_ < *r*_2_*a*_2_), the stability changes with *σ*. At lower values of *σ* the acidic equilibrium is locally stable, whereas at higher values of *σ* the opposite is true. Furthermore, the tendencies of the peak resilience (*R*_max_) and optimal composition (*X*_opt_) are qualitatively the same (electronic supplementary material, figure S5 in S2).

## 4. Discussion

The present theory proposes a fundamental mechanism for maintaining a microbial community. A general property of a bacterium, sensitivity to pH, invariably induces alternative stable states. When one population of bacteria dominates, the pH is biased to the optimum value of the dominant bacteria, which strongly inhibits the growth of another functional type of bacterium. Hence, microbiota can exist in alternative stable compositions. Furthermore, once one type of bacteria dominate, such an equilibrium can become more resilient to any disturbance if the microbial composition is balanced. In particular, when growth and the rate of change of pH exist in a trade-off relationship, resilience increased. We hypothesize therefore that an optimal level of pH sensitivity can create a balanced microbial composition that exists in a stable equilibrium.

Our model predicts that a microbial community is more resilient when growth and the rate of change of pH exist in a trade-off relationship. Specifically, the model requires that acidophilic bacteria are inferior in growth but superior in their ability to vary the rate of the change of pH. This is explained by a property of the equilibrium presented here, in which acidophilic bacteria were more abundant than alkaliphilic bacteria. Once the system reached such an equilibrium, a higher equilibrium population of acidophilic bacteria was probably maintained by changing the pH of their environment to the optimum value. By contrast, a lower equilibrium population of alkaliphilic bacteria was probably maintained by its rapid growth. This rapid recovery mechanism may help maintain microbial communities. By contrast, the mechanism should not work in another equilibrium comprising a larger abundance of alkaliphilic bacteria, because the trade-off conversely weakens resilience, suggesting that the alkaline equilibrium can be less resilient. Actually, we can show that resilience was always higher in the acidic equilibrium (electronic supplementary material, S1). If so, once a strong perturbation shifts the equilibrium to an alkaline state, it may be recovered with relative ease. The trade-off between growth and the rates of pH change may play a key role in maintaining microbial communities.

The resilience of an ecosystem may relate to its microbial composition. The present theory suggests that resilience is high when the microbial composition is balanced. In other words, microbial compositions may frequently change if the ecosystem comprises imbalanced microbial composition. A possible test of this hypothesis is to compare microbial compositions between different communities under varying levels of disturbance. An ecosystem under more intense disturbance is expected to have more stably balanced composition. Although we can hardly discuss intestinal flora without accounting for its relationship with the host, it has been suggested that a healthy intestinal flora population can maintain itself [36]. Incorporating interactions with hosts into the model is the next important step for understanding the relationship between microbial composition and host health.

A recent study proposed a similar model to our own using a bell-shaped function for bacterial growth [34]. In the other model, bistability occurs. However, in an equilibrium, one type persists and the other goes extinct, which is contrary to that observed in our system. This difference arises from assumptions regarding pH effects on bacterial growth. It was assumed that pH affects overall bacterial growth, including the self-regulation term [34]. This implies that bacteria do not grow once the pH environment changes to a non-preferred pH environment. As this assumption critically alters the behaviour of the system, careful examination of the effects of pH on bacteria is warranted.

Our conceivable, simple model of an ecosystem of microbial communities presented here assumes that bacteria form two functional groups. In real microbial communities, however, various types of microbes coexist [34,35]. In other bacteria, the pH that is preferred and changed by them is different. For instance, *Pseudomonas veronii* increases the pH but prefers low pH values for growth. *Serratia marcescens* lowers the pH but prefers high pH values [34,37]. A recent study showed that such bacteria can cause self-extinctions [34,35]. However, in real ecosystems, diverse microbes coexist. Hence, it remains an open question as to how multiple bacteria coexist in complex ecosystems and affects multistability [15].

## Supplementary Material

Mathematical analysis

## Supplementary Material

Supplemental figures

## Supplementary Material

Mathematica code that can produce the all figures

## Data accessibility

Mathematica code supporting the results in this study is available as electronic supplementary material (S3).
