# Supplementary material for: A coexistence theory in microbial communities
Source: R Soc Open Sci. 2018 Sep 19;5(9):180476. doi: 10.1098/rsos.180476 (PMC6170546; doi:10.1098/rsos.180476)
Supplement: Mathematical analysis [file rsos180476supp1.pdf]

Electronic Supplementary Material (S1)

Mathematical analysis

Local stability analysis of the coexistence equilibrium

By setting the right-hand sides of Eqs. 1a–c in the main text to zero, three nontrivial equilibria (X_1*, X_2*, Y*) are obtained as follows:

(Eq-i): $(r_1/(1 + e^{-\theta}), r_2/(1 + e^{\theta}), -1)$ (1a)

(Eq-ii): $(r_1/(1 + e^{\theta}), r_2/(1 + e^{-\theta}), 1)$ (1b)

(Eq-iii): $(a_2r_1r_2/(a_1r_1 + a_2r_2), a_1r_1r_2/(a_1r_1 + a_2r_2), \ln[r_1a_1/r_2a_2]/\theta)$ (1c)

We note that the last equilibrium requires $\theta \neq 0$, meaning that the preference for a particular pH value is required for this system.

Using local stability analysis, the stability of the coexisting equilibria can be assessed. We can judge local stability if the characteristic equation of their Jacobian matrix satisfies the Routh–Hurwitz criteria [1]. Under each equilibrium condition, we obtained the Jacobian matrixes. The characteristic equation for determining the eigenvalues is $\lambda^3 + w_1\lambda^2 + w_2\lambda + w_3 = 0$. The equilibrium is locally stable if $w_1 > 0$, $w_3 > 0$, and $w_1w_2 > w_3$. In each equilibrium (1a–c), we obtained the stability conditions as follows:

(i): $\theta > -\hat{\theta}$ (2a)

(ii): $\theta > \hat{\theta}$ (2b)

(iii$_a$): $\theta < \hat{\theta}$ (if $r_1a_1 > r_2a_2$) (2c-1)

(iii$_b$): $\theta < -\hat{\theta}$ (if $r_1a_1 < r_2a_2$) (2c-2)

where $\hat{\theta} = \ln[r_1a_1/r_2a_2]$.

Resilience of the equilibrium

When the equilibrium is locally stable, resilience, defined as the capacity of the system to return to equilibrium after a short and small disturbance, was calculated as the absolute value of the highest real part of eigenvalues of the Jacobian matrix [2]. Here we focus only on the first equilibrium (1a), because the same property is that of the second equilibrium, and the stable condition of the third equilibrium is limited (note that dynamics can converge on the third equilibrium under extreme initial values of pH, which favor one type of bacterium, because of the extreme value of the pH at equilibrium).

In the first equilibrium, we explicitly obtain the eigenvalues as follows:

(E-1) $2(r_2a_2 - r_1a_1e^{\theta})/(1 + e^{\theta})$ (3a)

(E-2) $-r_1/(1 + e^{-\theta})$ (3b)

(E-3) $-r_2/(1 + e^{\theta})$ (3c)

We find three distinct cases of maximum dominant eigenvalue (largest resilience, $R_{\max}$) along θ (Figs, S3 and 2). In each case, we can also calculate the optimal value of θ (θ_{opt}) at which resilience is largest. Each value $\{\theta_{\text{opt}}, R_{\max}\}$ is calculated as follows:

I) $\{\approx 0, \approx r_2/2\}$ (4a)

II) $\{\ln[r_2(2a_2 + 1)/2r_1a_1], 2a_1r_1r_2/(2a_2r_2 + r_2 + 2a_1r_1)\}$ (4b)

III) $\{\ln[r_2/r_1], r_2r_1/(r_2 + r_1)\}$ (4c)

The conditions of each case are obtained by both positional relationships in eigenvalues on the intercept ($\theta = 0$) and intersection points of eigenvalues. In case I, on the intercept, E-1 < E-3 and E-2 < E-3 are held. In case II, on the intercept, E-1 is larger than E-3, and an intersection point of eigenvalues E-1 and E-3 is larger than that of E-1 and E-2. In case III, on the intercept, E-2 > E-3, and an intersection point of eigenvalues E-1 and E-3 is smaller than that of E-1 and E-2. These conditions are as follows:

I) $r_2/r_1 < 1$ and $r_2/r_1 < 2a_1/(1 + 2a_2)$ (5a)

II) $r_2/r_1 > 2a_1/(1 + 2a_2)$ and $a_1 - a_2 < 1/2$ (5b)

III) $r_2/r_1 > 1$ and $a_1 - a_2 > 1/2$ (5c)

In each case, we can also calculate the ratios of equilibria of bacterial abundance ($X_{\mathrm{opt}} = X_1^*/X_2^*$ at the optimal θ_{opt}) by incorporating θ_{opt} into X_1^*/X_2^* (= $e^{\theta}r_G/r_B$):

I) $\approx r_1/r_2$ (6a)

II) $(2a_2 + 1)/2a_1$ (6b)

III) 1 (6c)

Here, we compare $R_{\max}$ in three cases (4). As shown in Fig. 3a, $R_{\max}$ of case III tends to be higher. Under the condition of case III ($r_2 > r_1$), by assuming $r_2 = r_1 + \Delta$ (Δ is a positive constant),

R_{max} in case III is rearranged as $(r_1 + \Delta)r_1/(2r_1 + \Delta)$. In contrast, under the condition of case I ($r_2 < r_1$), by assuming $r_2 = r_1 - \Delta'$ (Δ' is a positive constant), R_{max} in case I is rearranged as $(r_1 - \Delta')/2r_1$. Since $(r_1 + \Delta)r_1/(2r_1 + \Delta) > (r_1 - \Delta')/2r_1 \Leftrightarrow r_1\Delta > -(2r_1\Delta' + \Delta\Delta')$, R_{max} in case III is always higher compared with that of case I.

Next, consider the comparison between cases II and III. Under the condition of case II, because $a_1 - a_2 < 1/2$, here we assume $a_2 = a_1 - 1/2 + \mathrm{M}$ (where M is a positive constant). Then, R_{max} in case II is rearranged as $r_2r_1/\{r_2(1 + \mathrm{M}/a_1) + r_1\}$. Since $r_2r_1/\{r_2(1 + \mathrm{M}/a_1) + r_1\} < r_2r_1/(r_2 + r_1) \Leftrightarrow \mathrm{M}/a_1 > 0$, R_{max} in case III is always higher compared with that of case II.

We can compare R_{max} between alternative stable equilibria. We focus on case III in the above equilibrium (1a). Then we can calculate R_{max} in another equilibrium (1b) as $2a_2r_1r_2/(2a_2r_2 + r_1 + 2a_1r_1)$. The comparison of the two R_{max} values can show that resilience is always higher in the focusing equilibrium (1a) compared with that of another equilibrium (1b). If we assume $r_2r_1/(r_2 + r_1) > 2a_2r_1r_2/(2a_2r_2 + r_1 + 2a_1r_1)$, the inequality is rearranged to $1 + 2a_1 > 2a_2$, which is always true under the condition of case III, $a_1 - a_2 > 1/2$.

Analysis of an alternative model

We also considered another model with a bel-shaped function of pH effect on bacterial growth. The system is described by the following ordinary differential equations.

$$\frac{dX_1}{dt} = \left(r_1 e^{\frac{-(Y-p_1)^2}{\sigma^2}} - X_1 \right) X_1, \quad (7a)$$

$$\frac{dX_2}{dt} = \left(r_2 e^{\frac{-(Y-p_2)^2}{\sigma^2}} - X_2 \right) X_2, \quad (7b)$$

$$\frac{dY}{dt} = \left(a_2 X_2 - a_1 X_1\right)\left(1 - Y^2\right), \quad (7c)$$

where p_i is the optimal pH for each bacteria ($p_1 < 0$ and $p_2 > 0$) and σ is the pH sensitivity parameter.

First, we find that the two alternative non-trivial equilibria, (X_1*, X_2*, Y*): (Eq-1) $\left(r_1 e^{(1+p_1)^2/\sigma^4}, r_2 e^{-(1+p_2)^2/\sigma^2}, -1\right)$ and (Eq-2) $\left(r_1 e^{-(1-p_1)^2/\sigma^2}, r_2 e^{-(-1+p_2)^2/\sigma^2}, 1\right)$. Because of the same procedure, we skip the details of local stability analysis, Then, we could obtain the local stability condition in the first equilibrium:

$$\ln[r_2 a_2 / r_1 a_1] < (1 + p_1)^2/\sigma^4 + (1 + p_2)^2/\sigma^2 \quad (8)$$

The direct simulation confirms that the stability changes and switches to alternative equilibrium (Fig. S4). Furthermore, by numerical analysis, we also confirmed that the maximum resilience R_{max} and optimum microbial composition X_{opt} in the first equilibrium show qualitatively same patterns as the main model (Fig. S5).

References

1. May RM (1974) Stability and complexity in model ecosystems (Princeton Univ. Press: Princeton, NJ).
2. Pimm SL, Lawton JH (1977) Number of trophic levels in ecological communities. *Nature* 268:329-331.
