# Supplementary material for: A coexistence theory in microbial communities
Source: R Soc Open Sci. 2018 Sep 19;5(9):180476. doi: 10.1098/rsos.180476 (PMC6170546; doi:10.1098/rsos.180476)

Electronic Supplementary Material (S2)

Supplemental figures

Fig. S1. Dependence of the initial microbial composition on in the final compositional state. (a–e) $r_1a_1 > r_2a_2$. We assumed $r_1 = 2$, $r_2 = 1$, $a_1 = 1.5$ and $a_2 = 1$. (f–j) $r_1a_1 < r_2a_2$. We assumed $r_1 = 1$, $r_2 = 2$, $a_1 = 1$ and $a_2 = 1.5$. Blue and red areas indicate the cases where the dynamics reach to the equilibria dominated by the acidophilic and alkaliphilic bacteria, respectively.

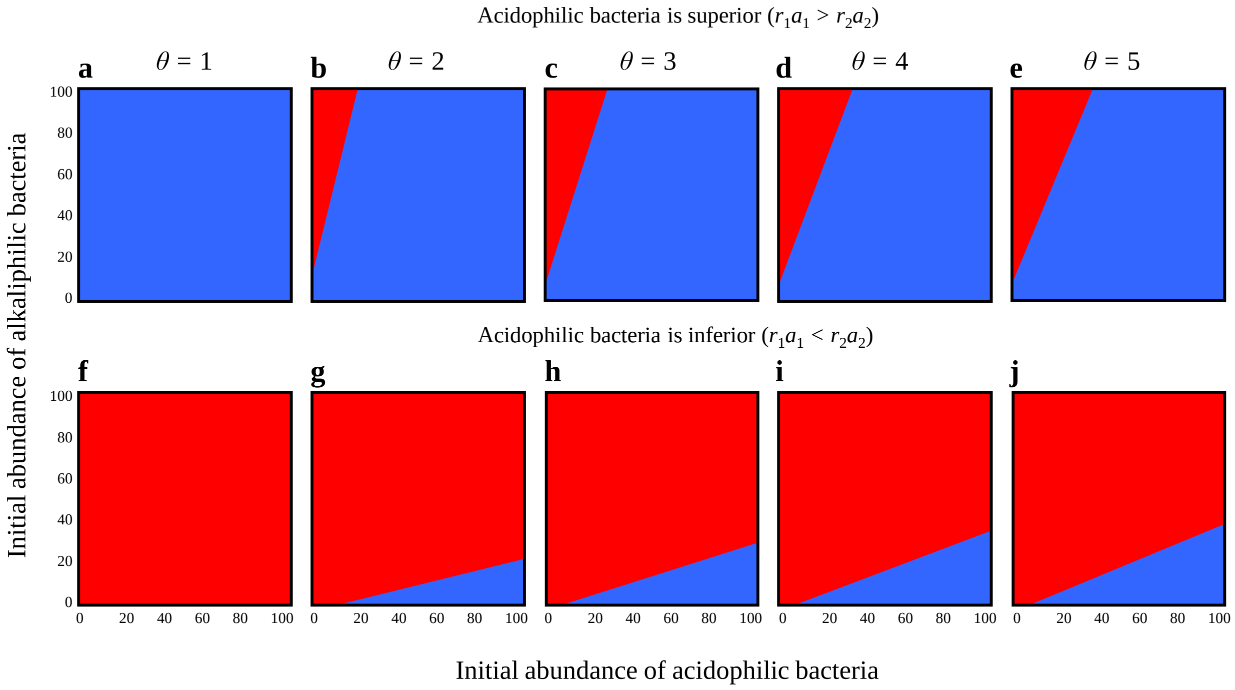

Fig. S2. Optimal microbial composition associated with peak resilience. We consider case II in Fig. 2. X_1*/X_2* at the peak of resilience, $(2a_2+1)/2a_1$. Case II is constrained within the white region (S1 in ESM).

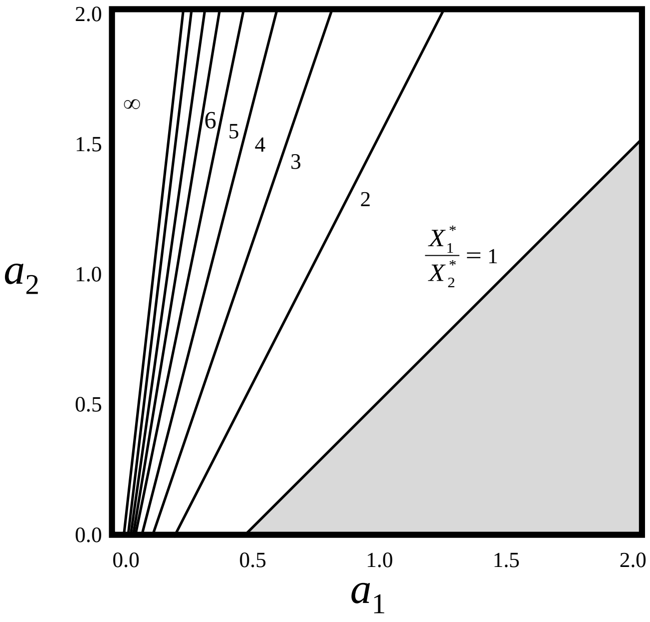

Fig. S3. Typical cases of shifts of dominant eigenvalues as a function of θ. Different colors indicate the differences in eigenvalues (3a–c in S1 in ESM). In the gray regions, the equilibrium is locally unstable. Parameters (r_1, r_2, a_1, a_2) in (a–g) are as follows: (2, 1, 2, 1), (2, 1, 1, 1.8), (1, 2, 1, 2), (1.5, 2, 2, 1), and (1, 2.5, 2.5, 1.5), respectively.

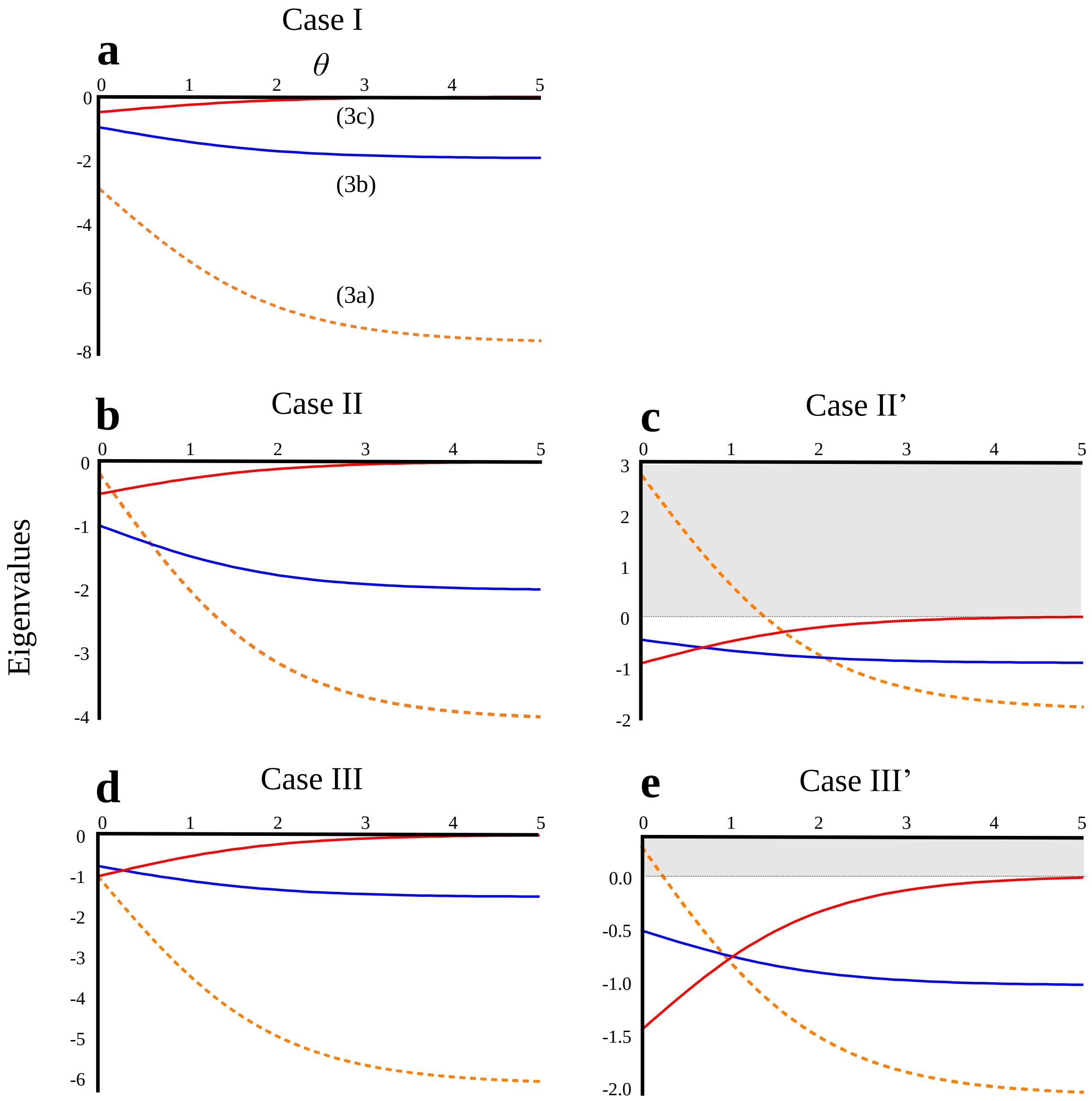

Fig. S4. Equilibrium abundance of acidophilic bacteria varies with sensitivity to pH in the model with a bell-shaped pH sensitivity. (a) $r_1a_1 > r_2a_2$. (b) $r_1a_1 < r_2a_2$. The equilibria are obtained by direct simulations. Depending on the initial abundances, the system can converge to either more abundant or less abundant equilibrium. The bistability occurs when pH sensitivity is less than a threshold. Arrows indicate the directions of system behavior. $\hat{\sigma}$ indicate the threshold value of σ at which the stability shifts. We assumed that $p_1 = -1$ and $p_2 = 1$. In (a), $r_1 = 1$, $a_1 = 1.1$, $r_2 = 1$, and $a_2 = 1$. In (b), $r_1 = 1$, $a_1 = 1$, $r_2 = 1$, and $a_2 = 1.1$.

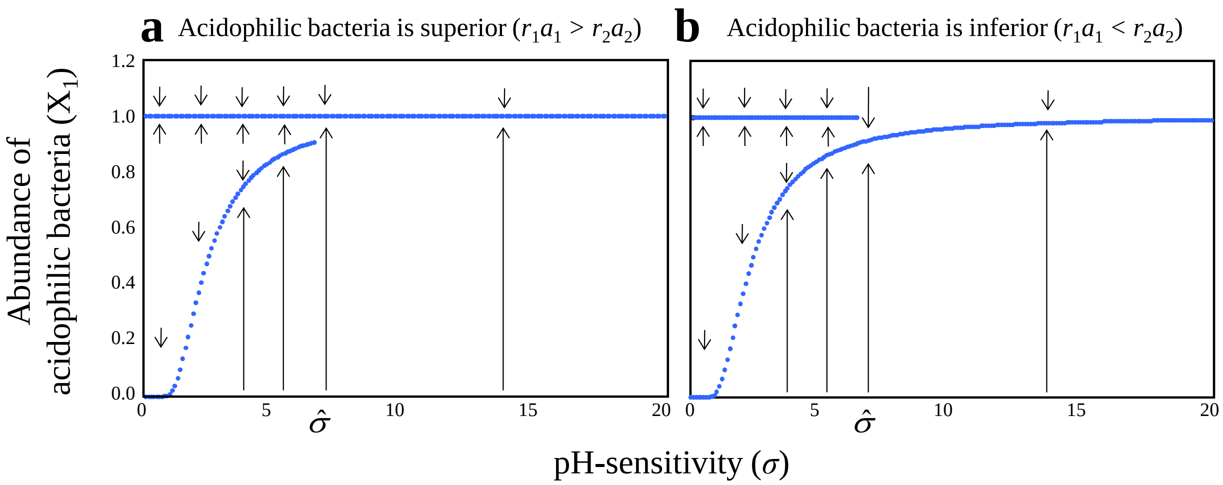

Fig. S5. Maximum resilience (a) and optimum microbial composition (b) in the first equilibrium in the model with a bell-shaped pH sensitivity. We assumed $p_1 = -1$, $p_2 = 1$, and $r_1 = a_1 = 1$. Contours in (a) and (b) indicate the values of R_{max} and X_{opt}, which are numerically obtained. The position of $r_1 = a_1$ (= 1) is indicated by the yellow circles.

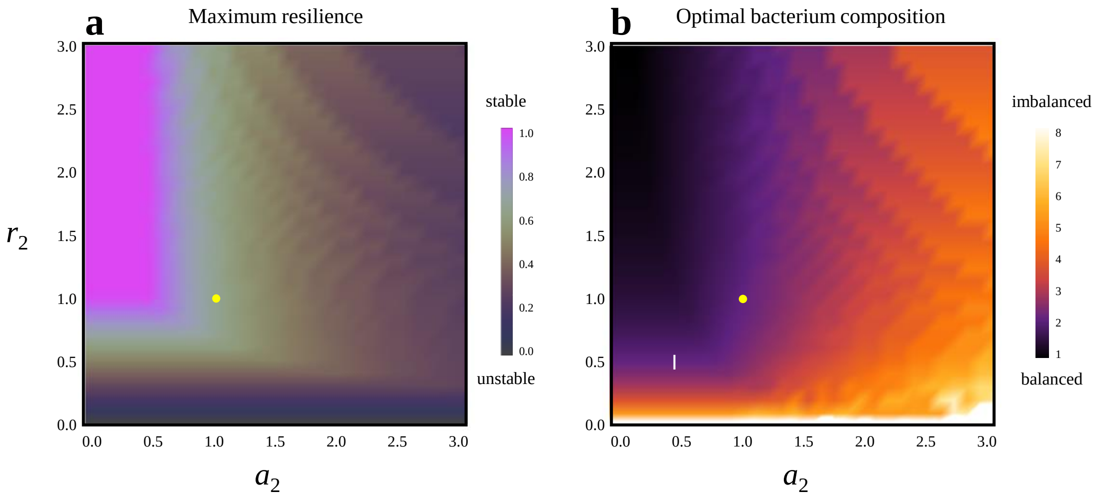

Supplement: Supplemental figures [file rsos180476supp2.pdf]
